# Supplementary material for: Loss of BICD2 in muscle drives motor neuron loss in a developmental form of spinal muscular atrophy
Source: Acta Neuropathol Commun. 2020 Mar 17;8:34. doi: 10.1186/s40478-020-00909-6 (PMC7076953; doi:10.1186/s40478-020-00909-6)
Supplement: Supplementary file 1 — Additional file 1: Supplementary Figures. [file 40478_2020_909_MOESM1_ESM.docx]

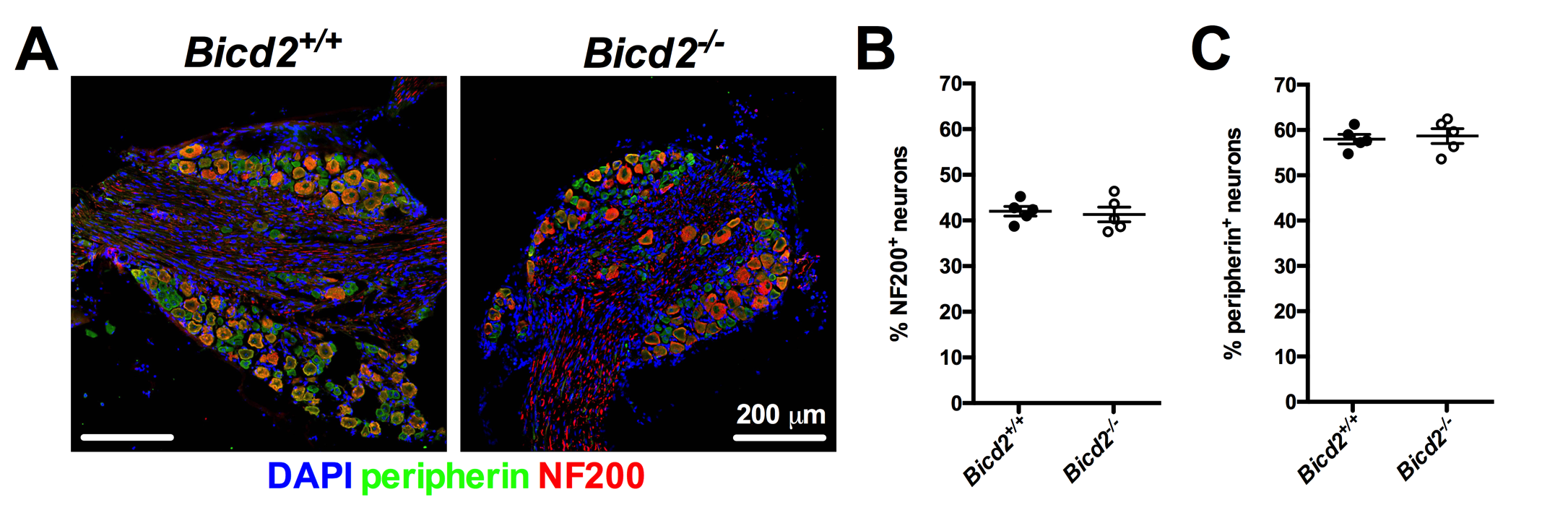


**Supplementary Figure S1**. There is no difference in the percentages of medium-to-large (NF200^+^) and small (peripherin^+^) sensory neurons between *Bicd2^+/+^* and *Bicd2^-/-^* mice. (**A**) shows representative images of L4 dorsal root ganglia stained for DAPI (blue), peripherin (green) and NF200 (red). (**B** & **C**) There is no difference in the percentages of NF200^+^ or peripherin^+^ sensory neurons between *Bicd2^+/+^* and *Bicd2^-/-^* mice (NF200, *Bicd2^+/+^* 42% ±1 (n=5), *Bicd2^-/-^* 41% ±1.6, unpaired *t*-test p=0.73, peripherin, *Bicd2^+/+^* 58% ±1 (n=5), *Bicd2^-/-^* 59% ±1.6, unpaired *t*-test p=0.73).


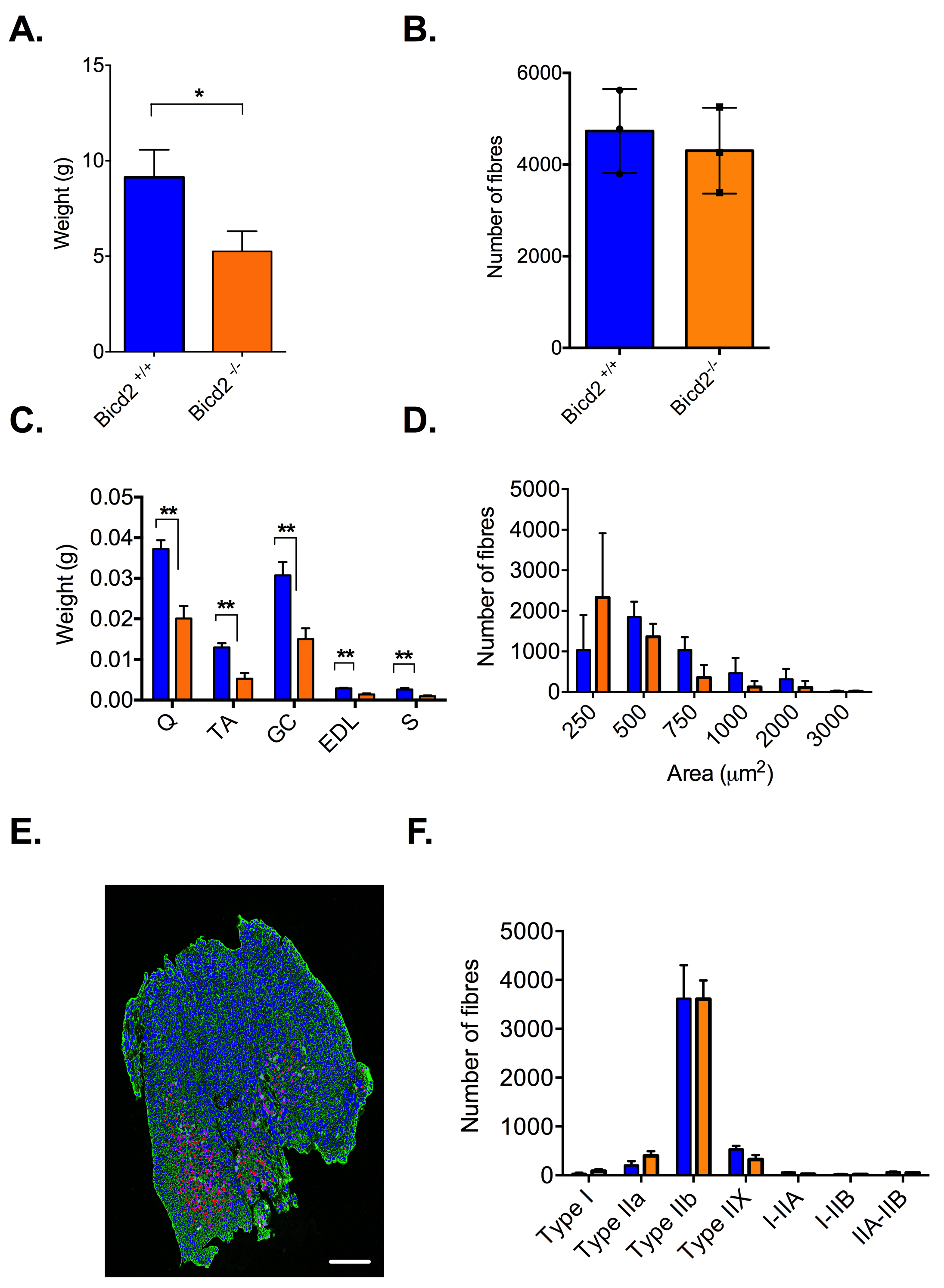


**Supplementary Figure S2**. A quantitative comparison of the gastrocnemius muscle in *Bicd2^+/+^* and *Bicd2^-/-^* mice at 21 days of age. (**A**) shows the weight in grams of *Bicd2^+/+^* (n=5) and *Bicd2^-/-^* (n=4) mice, **p*=0.0028 (unpaired *t*-test). (**B**) shows the number of muscle fibres of the gastrocnemius muscle in *Bicd2^+/+^* (n=3) and *Bicd2^-/-^* (n=3) mice. (**C**) shows a comparison of the weight in grams in the muscles of *Bicd2^+/+^* (blue, n=3) and *Bicd2^-/-^* (orange, n=3) mice. Q=quadriceps, TA=tibialis anterior, GC=gastrocnemius, EDL=extensor digitorum longus, S=soleus, ***p*<0.01 (multiple t-tests corrected for multiple comparisons using the Holm-Sidak method). (**D**) is a histogram of the gastrocnemius muscle fibres according to diameter (blue=*Bicd2^+/+^*, orange=*Bicd2^-/-^*). (**E**) shows a digital reconstruction generated from images presented in **Supplementary Figure 3F** following automated fibre type quantification using an ImageJ plugin called ‘muscle-J’. Red = type 1, magenta = type IIA, dark blue = type IIX, light blue = type IIB). (**F**) shows the mean number of muscle fibre types in the gastrocnemius muscle of *Bicd2^+/+^* (n=3) and *Bicd2^-/-^* (n=3) mice. Error bars = SEM.

**
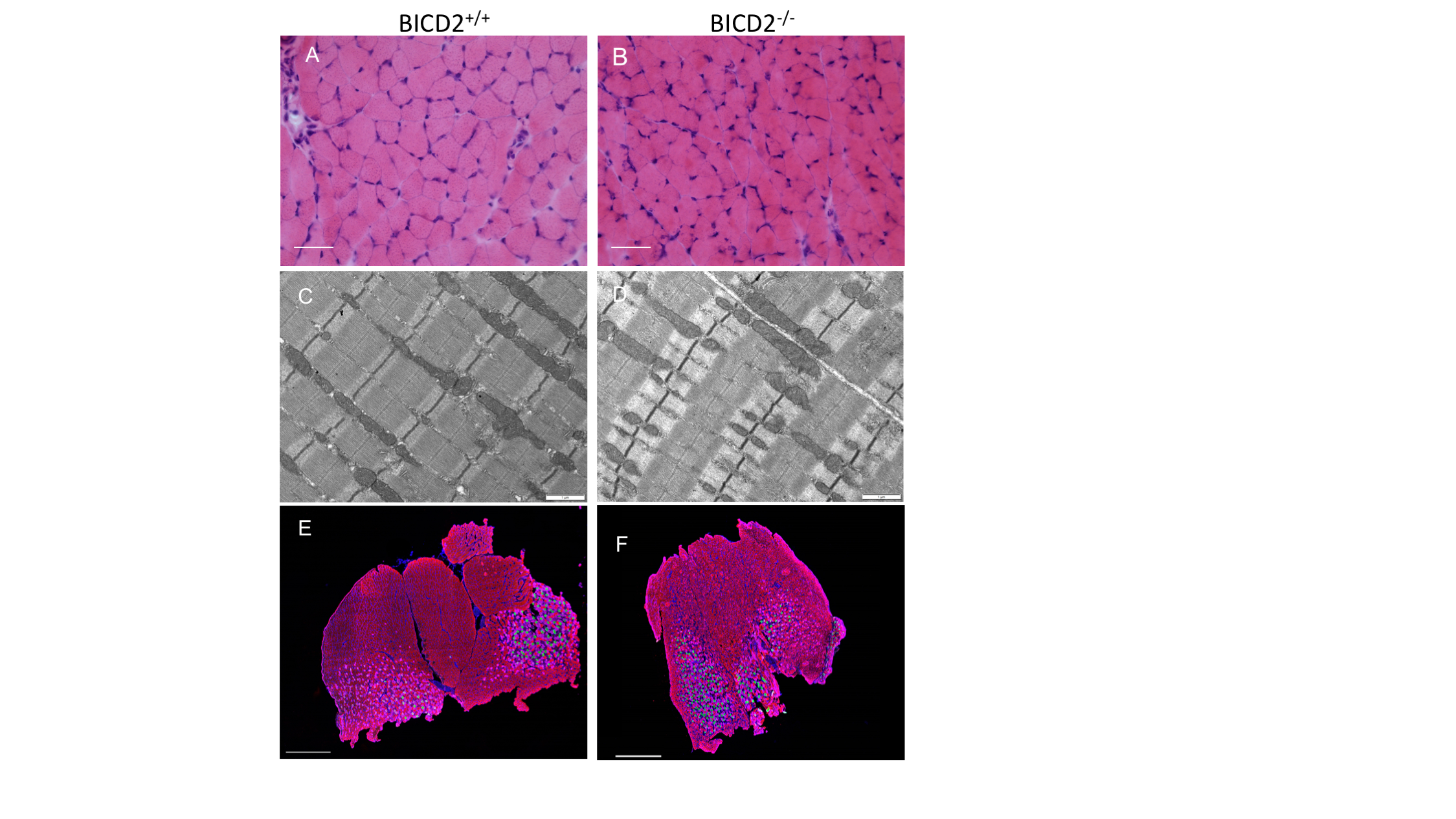
**

**Supplementary Figure S3**. No histological differences between *Bicd2^+/+^* and *Bicd2^-/-^* gastrocnemius muscle at 21 days of age. Haematoxylin and eosin stains of the gastrocnemius muscle of *Bicd2^+/+^* (**A**) and *Bicd2^-/-^* (**B**) mice, scale bars = 50 µm. Transmission electron microscopy image of the gastrocnemius muscle in *Bicd2^+/+^* (**C**) and *Bicd2^-/-^* (**D**) mice. Scale bars = 1 μm. (**E** & **F**) show immunohistochemical staining for muscle fibre types in 10 µm cryosections of the gastrocnemius muscle in *Bicd2^+/+^* and *Bicd2^-/-^* mice, respectively. Green= type 1, pink = type IIa, red = type IIb, no staining = type IIx, blue = laminin, scale bars = 500 µm.
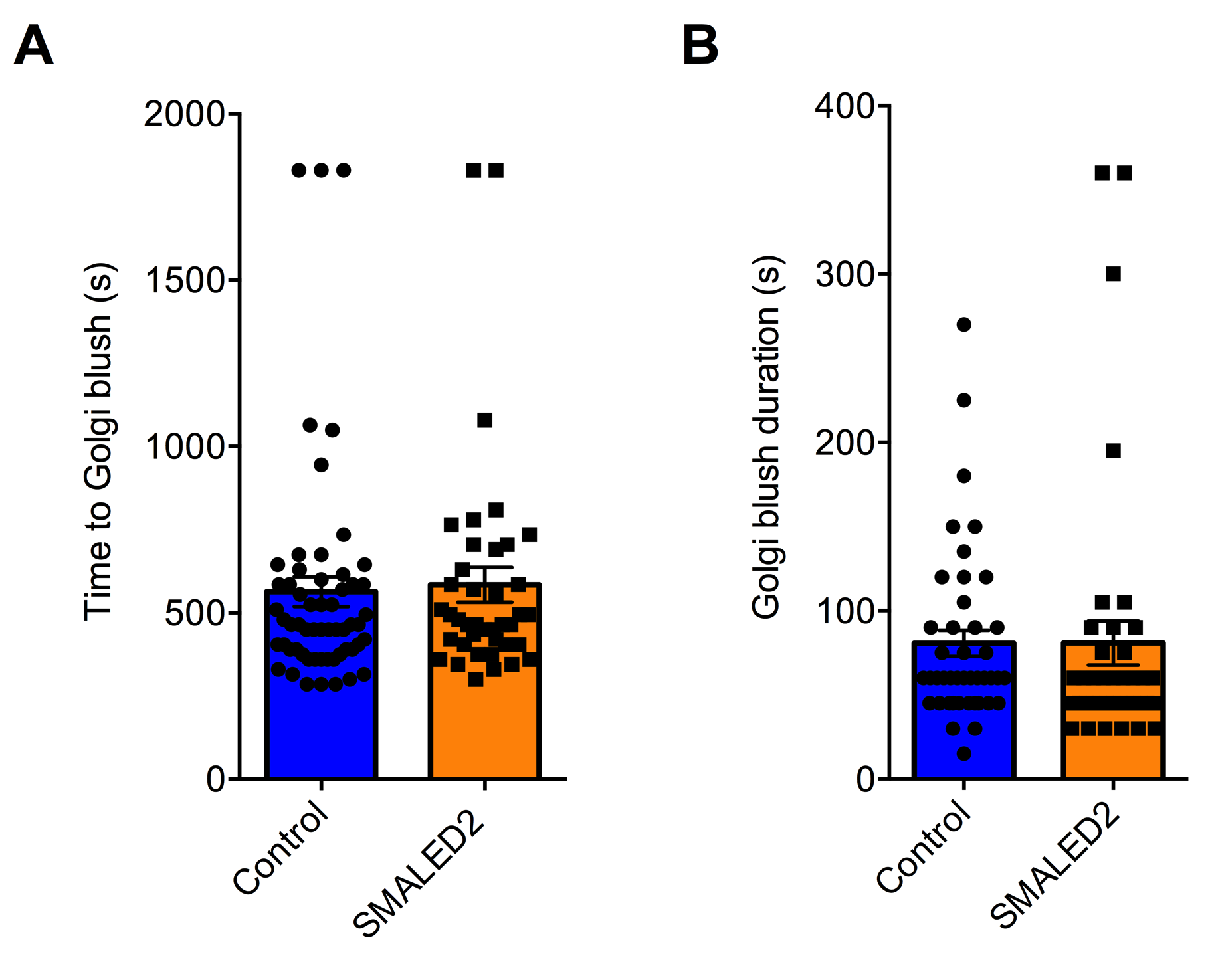


**Supplementary Figure S4**. Human fibroblasts from a healthy control (blue) and SMALED2 patient (I189F mutation, orange) were plated on to 20 mm coverslips in 6-well plates at a density of 100,000 cells per well. After 24 h in culture, cells were transfected with a plasmid expressing human galactosyltransferase fused to GFP (GT–GFP). Golgi were identified by GFP staining. Time-lapse confocal imaging was performed every 5 s, and the time taken from the addition of Brefeldin A to the beginning and end of the Golgi blush recorded. The Golgi blush describes the appearance of the Golgi as it loses structure and is resorbed into the ER. (**A**) shows the time taken from the addition of Brefeldin A to the start of the Golgi blush (control, median time = 465 s (n=58 cells), SMALED2 = 473 s (n=40 cells), Mann-Whitney *U* test, *p*=0.44) and (**B**) shows the duration of the Golgi blush (control, median time = 60 s, n=43 cells; SMALED2 = 60 s, n=39 cells; Mann-Whitney *U* test, *p*=0.13). Both parameters are used as surrogate markers of transport from the cis-Golgi apparatus to the ER.
